# The Book World of Medicine and Science

**Published:** 1895-07-06

**Authors:** 


### THE BOOK WORLD OF MEDICINE AND SCIENCE.

THE PREVENTION OF SMALL-POX. By EDGAR M. CROOKSHANK, M.B. (London: K. Lewis. 1894. Pp. 40. Price 1s.)

This is a reprint of an inaugural address given at King's College at the commencement of the winter session. It is more especially devoted to an account of the origin and development of the stamping-out system. A sketch of the history of the malady is given, and it is shown that for many years the large confidence which the profession had in vaccination as a preventative of the disease led to an unjustifiable neglect of such measures as isolation; but it is pointed out that by means of the Notification Act and the large number of hospitals which have been established during recent years, and the still larger number which we may hope to see established under the provisions of the Isolation Hospitals Act of 1893, a very efficient machinery is put into our hands, by which it may be expected that the disease may be kept well under control.

OVARIAN NEURALGIA. By A. RABAGLIATI, M.A., F.R.C.S. Edin. (London: Ballière, Tindall, and Cox. 1895. Pp. 77.)

The above is the short title on the cover of the book. On the title-page, however, it takes a much more ponderous form, and runs as follows: "On some Symptoms which Simulate Disease of the Pelvic Organs in Women, and their Treatment by Allo-piesto-myo-kinetics (Massage) and by Auto-piesto-myo-kinetics (Self-movements of Muscles under Pressure)." Now, when an author begins by calling massage by such a portentous name, one is apt to put him down as a pedant, and to look for dulness in his book. In this case, however, our anticipations were not fulfilled, for, in fact, the work is rather interesting. It is well written, and, what is a mercy nowadays, it evidently expresses the firm convictions of the writer. The main drift and intentions of the book may be expressed in two sentences: "Generally speaking, a woman does not become ill because her uterus and generative apparatus is out of order, but her uterus is out of order because she is ill . . . I repeat that he who treats the uterus through the woman will do much better, and be much more successful, than he who treats the woman through the uterus." The work is, in fact, the outcome of a profound dissatisfaction with both of the two popular explanations of women's ailments, one of which puts them all down to uterus and the other to nerve. Unfortunately, Dr. Rabagliati does but add one more to the list of the all-embracing explanations to which the endless variety of women's ailments are sought to be brought into conformity. As fashion oscillates from uterus to nerve, our author seems to catch it in mid-air and tries to tie it down to muscle. The aches and pains and ailments of which women so constantly complain, are shown to have their seat in muscles and in muscle sheaths, and in the fibrous structures of the joints; it is argued that at the bottom of them all is general malnutrition. The treatment then becomes largely one of diet and of movement of the muscles—in some cases by massage, in others by active movements while the muscles which are being exercised are at the same time held or compressed. Various suggestions are made regarding the diet best suited for the conditions described, and details are given as to the exercises ordered. In regard to the latter, various photographs are appended to the book, which, while very life-like and, doubtless, very explanatory of the author's meaning, are not very attractive from an æsthetic point of view.

WALKS IN BELGIUM: THE OLD FLEMISH CITIES AND THE ARDENNES. Edited by PERCY LINDLEY. (London: 30, Fleet Street. Price 6d.)

A very suggestive little guide-book has been prepared by Mr. Percy Lindley on the region which he has made peculiarly his own, embracing the French, Belgian, and Luxemburg Ardennes. The key-note of the various tours, described with a double view to the picturesque and the practical, is economy, but it by no means follows that the traveller will not, with the small expenditure indicated, fare fully as well as if means were forthcoming for lengthy journeys and big hotels. Return tickets to Antwerp are issued viâ Harwich at 24s. second class, and not only is the railway travelling on the other side very cheap, but it is possible to travel third class as in England. Settling down in the Ardennes district, pension terms at from four francs a day can be arranged, and from almost any point walks of the most varied beauty and innumerable places of historical interest are within reach. The pedestrian notes with the excellent maps and plans comprise all the information required to guide the most inexperienced in choosing headquarters and carrying out plans; and the numerous sketches of the country help to make up a pretty and entertaining as well as very useful little volume.

